# Fractures of the Sternum

**Published:** 1858-02

**Authors:** Frank Hastings Hamilton


### ART. II.—*Fractures of the Sternum.* By FRANK HASTINGS HAMILTON, M. D.

Fractures of the sternum are of rare occurrence, owing, probably, to the elasticity of the ribs and their cartilages, upon which it mainly rests, and also in part, to the softness of its structure. In advanced life, the ossification and fusion of all of its several portions becoming more complete, and the cartilages of the ribs also becoming more or less ossified, its fracture is relatively, more frequent.

*Causes.* They are generally the result of direct blows inflicted upon the part, such as the passage of a loaded vehicle across the chest, the fall of a tree or of some heavy timber upon the body; the fracture implying, always, that great force has been applied.

Indirect blows, and voluntary muscular action alone have been known also occasionally to produce this fracture.

David, in his Mémoire sur les Contrecoups, published as a prize essay by the Academy of Medicine, mentions the case of a mason, who, in falling from a great height, struck upon his back against a cross-bar which intercepted his fall, in consequence of which the abdominal and sterno-cleido-mastodian muscles were so stretched as that the sternum broke asunder between its upper and middle portions.* Sabatier reports another case of fracture at the same point, produced in a similar manner;† and Rolland has described

---

* Boyer ou Bones, p. 57.

† Malgaigne, from Sabatier, Mem. sur la Fract. du Sternum.

a third example in a woman sixty-three years old, who, falling from a height backwards and striking upon her back, broke the sternum near its centre.*

Cruveilhier saw a man who having fallen from a height of twenty feet upon his nates was found to have a fracture of the sternum.† Cussan saw the same result in a person who fell from a third story, striking first upon his feet and then pitching over upon his back.‡ Maunoury and Thore have reported an analagous case, where a man fell from a height of twelve or fifteen *mètres*, first striking upon his feet and then falling over upon his back and head.§ Mr. Johnson, late editor of the London Med. Chi.-Rev., reports a case of this kind, also, as having been received into St. George's Hospital, in London; the man, a healthy laborer, from the country, had fallen from the top of a hay cart, striking only upon his head. He walked with his head much bent forwards, and was incapable of either flexing, extending or rotating it any farther. The fracture was transverse, and about three inches below the top of the sternum opposite the centre of the third rib, the lower fragment projecting in front of the upper. The fragments were easily replaced by simply throwing the head back, and they fell into place with an audible snap, but they immediately resumed their unnatural position when the head was flexed. They finally united, but with a slight projection and overlapping.‖

Malgaigne expresses a doubt whether all these can be considered as the results of muscular action, since in a certain number of the examples cited, the head seems to have been thrown forwards by the concussion, and in others, also, there is no evidence that the muscles attached to the sternum were put upon the stretch. The only remaining explanation is that in such cases the sternum has been broken by the violent shock, or *contrecoup*.

*Seat and Direction of Fracture.* The sternum is separated most frequently either in the long central portion, or at the junction of this latter with the upper portion where the bone is weakest. In fact a separation at this latter point may be regarded frequently as a diastasis or dislocation rather than as a fracture, since the two portions do not become firmly united by bone until late in life. The very late ossification and fusion of the

---

* Malgaigne, from Bull. de Thér., tom. vi, p. 288.
† Malgaigne, from Bull. de la Soc. Anat., juin, 1826.
‡ Malgaigne, from Archiv. de Méd., janv., 1827.
§ Malgaigne, from Gaz. Méd., 1842, p. 361.
‖ London Med. Chir. Rev., vol, xvii. new series, p, 536. 1832.

zyphoid cartilage with the central piece, also, will explain the infrequency of its fracture.

Boyer believed that the zyphoid cartilage was not susceptible of being permanently displaced backwards, except in aged persons after it had become ossified, "for," he says, "though violently struck and driven backwards by a blow on what is vulgarly termed the pit of the stomach, yet it restores itself by its own elasticity."*

The following case, however, which has come under my own observation, is conclusive as to the possibility of this accident:

A man, twenty-eight years old, fell forwards striking the lower end of his sternum upon the top of a candlestick, breaking in the zyphoid cartilage. During two years following the accident he had frequent attacks of vomiting, which were excessively violent and distressing. The paroxysms occurring every five or six days. Both Dr. Green, of Albany, and Dr. White, of Cherry Valley, upon whom he called for relief, recommended excision of the bone, but the patient would not submit to the operation. Twelve years after the accident, in the year 1848, while he was an inmate of the Buffalo Hospital of the Sisters of Charity, I examined his chest and found the zyphoid cartilage bent at right angles with the sternum, pointing directly towards the spine. He now suffered no inconvenience from it except that it hurt him occasionally when he coughed.†

The upper portion of the sternum is rarely broken, unless at the same time the central portion is broken also.

The direction of these fractures is generally transverse, or nearly so; occasionally a slight obliquity is found in the direction of the thickness of the bone. In three or four examples upon record the direction of the fracture was longitudinal. It is not so infrequent, however, to find the bone comminuted. Compound fractures are exceedingly rare.

When the fracture is transverse, the lower fragment is almost always displaced forwards, and some times it slightly overlaps the upper fragment.

In one instance mentioned by Sabatier, where the separation had taken place at the point of junction between the first and second piece, the lower fragment was displaced backwards, and was also carried upwards under the upper fragment to the extent of twenty-eight *millimétres.*

*Diagnosis.* In a few cases the patients have felt the bone break at the

---

* Boyer, on Diseases of Bones, p. 59.

† This Journal, vol. xii, p. 282, Cases of Fractures of the Sternum.

moment of the accident. When displacement exists it may generally be easily recognized, and the lower fragment will often be seen to move forwards and backwards at each inspiration and expiration. Crepitus may also be detected in some of these examples, but it is less often present where no displacement exists. To determine the existence of crepitus the hand should be placed over the supposed seat of fracture, while the patient is directed to make forced inspirations and expirations, or the ear may be applied directly to the chest.

Emphysema has, also, occasionally been noticed, indicating usually that the lungs have been penetrated by the broken fragments.

The frequent occurrence of congenital malformations of the sternum should warn us to exercise great care in our examinations lest we mistake these natural irregularities for fractures. Bransby Cooper mentions a remarkable instance of malformation of the zyphoid cartilage which he at first suspected to be a fracture. It was so much curved backwards that, as Mr. Cooper thinks, its pressure upon the stomach produced a constant disposition to vomit whenever he had taken a full meal, or had taken a draught of water.*

*Prognosis.* In simple fracture of this bone, uncomplicated with lesions of the subjacent viscera, and especially where the fracture is the result of muscular action or of a counter stroke, no serious consequences are to be apprehended. The bone unites promptly even where it is found impossible to bring their broken edges into apposition. Indeed, generally, where the fragments have been once completely displaced although it is not difficult to replace them momentarily, a redisplacement soon occurs, and they are found finally to have united by overlappings: but no evil consequences usually result from this malposition. In nearly all of the cases reported in which palpitations, difficult breathing, &c., have been charged to the persistence of the displacement, the injuries were of such a character as to furnish for these unfortunate results other and much more adequate explanations. In one instance, only, already mentioned, serious inconveniences followed from a displacement of the cartilage backwards.

In other cases, however, where the fracture is the result of a direct blow, constituting a large majority of the whole number, the prognosis is often very grave: a conclusion to which one would naturally arrive from the fact already stated, that the fracture of the sternum thus produced, in itself implies the application of great force.

---

* B. Cooper, Princ. and Prac. of Surg., p. 359.

An abscess occurring in the anterior mediastinum, and caries or necrosis of the bone are among the most common results of a blow delivered directly upon the sternum; complications which generally end sooner or later in death. Blood may be also extensively effused into the anterior mediastinum.

Where emphysema is present we may anticipate inflammation of the pleura and of the lungs.

In several instances, where death has occurred speedily after the injury, the heart has been found penetrated and torn by the fragments. Sanson and Dupuytren have each reported one example of this kind. Duverney has mentioned two, and Samuel Cooper says there is a specimen in the museum of the University College, exhibiting a laceration of the right ventricle of the heart by a portion of fractured sternum. Watson mentions a case in which the pericardium was torn, but the heart was only contused.*

*Treatment.* When the fragments are not displaced, the only indications of treatment are to immobilize the chest, and to allay the inflammation, pain, &c., consequent upon the injury to the viscera of the chest. The first of these indications is accomplished, at least in some degree, by enclosing the body, from the arm pits down to the margin of the floating ribs, with a broad cotton or flannel band. A single band, neatly and snugly secured, and made fast with pins, is preferable to, because it is more easily applied than the roller which surgeons have generally employed; it is also much less liable to become disarranged. It should be pinned while the patient is making a full expiration. To prevent its sliding down, two strips of bandage should be attached to its upper margin and crossed over the shoulders in the form of suspenders.

Generally the patients prefer the half sitting posture, with the head and shoulders thrown a little backwards; and this is the position which will be most likely to maintain the fragments in place, and also to secure immobility to the external thoracic muscles, while it leaves the diaphragm and the abdominal muscles free to act.

The second indication may demand the use of the lancet; but more often it will be found necessary to allay the pain and disposition to cough by the use of opium.

If, however, the fragments are displaced, it is proper first to attempt their reduction; which, as we have already intimated, is generally more easy of

---

* New York Journal Med., vol. iii, p. 351.

accomplishment than is the maintenance of them in place until a cure is effected.

The upper fragment may be thrown forwards, and made to resume its position sometimes by a single full inspiration; but then it usually falls back during expiration: or it may be reduced by straightening the spine forcibly and at the same time drawing the shoulders back.

Verduc and Petit proposed, in those cases in which it was found impossible to reduce the fragments by these simple means, to cut down and lift the depressed bone. Nelaton suggests the use of a blunt crotchet introduced through a narrow incision; and Malgaigne has thought of another plan, which is, to penetrate the skin with a punch, and directing it to the broken margin, to push the fragment into its place, but which he does not himself regard as a suggestion of much value, since the bone is too soft to afford the necessary resistence; and, moreover, this in common with all of the other similar methods is liable, in some degree, to the objection that it may increase the tendency to caries and suppuration, already imminent. If reduced, the fragments will probably immediately again become displaced; and more than all, it still remains to be proven conclusively, that the mere riding of the fragments is in itself ever a cause of subsequent suffering or even of inconvenience.

When an abscess has formed in the anterior mediastinum surgeons have occasionally recommended the use of the trephine. Gibson has twice operated in this manner at the Philadelphia Hospital, but in each case the caries continued to extend and the patient died; an experience which has inclined him latterly to discountenance the operation.*

There are other considerations mentioned by Lonsdale, which ought to decide us never to use the trephine in these cases. "For the symptoms denoting the presence of the abscess, when completely confined to the under surface of the bone, will be very uncertain; and when the matter collects in larger quantities, it will show itself at the margin of the sternum, between the ribs; when it can be let out by making a puncture with the point of a lancet, without the necessity of removing a portion of the bone."†

---

* Gibson, Institutes and Practice of Surgery, vol. i, p. 269.

† Lonsdale. Practical Treatise on Fractures. London, 1838, p. 242.

